# A 3.3 kV SiC Semi-Superjunction MOSFET with Trench Sidewall Implantations

**DOI:** 10.3390/mi16020188

**Published:** 2025-02-06

**Authors:** Marco Boccarossa, Kyrylo Melnyk, Arne Benjamin Renz, Peter Michael Gammon, Viren Kotagama, Vishal Ajit Shah, Luca Maresca, Andrea Irace, Marina Antoniou

**Affiliations:** 1 School of Engineering, University of Warwick, Coventry CV4 7AL, UK; kyrylo.melnyk@warwick.ac.uk (K.M.); arne.renz@warwick.ac.uk (A.B.R.); p.m.gammon@warwick.ac.uk (P.M.G.); viren.kotagama@warwick.ac.uk (V.K.); vishal.shah@warwick.ac.uk (V.A.S.); marina.antoniou@warwick.ac.uk (M.A.); 2 Department of Electrical Engineering and Information Technologies, University of Naples Federico II, Via Claudio 21, 80125 Naples, Italy; luca.maresca@unina.it (L.M.); andrea.irace@unina.it (A.I.)

**Keywords:** SiC MOSFET, superjunction, semi-superjunction, trench etching, sidewall implantation, tilted trench

## Abstract

Superjunction (SJ) technology offers a promising solution to the challenges faced by silicon carbide (SiC) Metal Oxide Semiconductor Field-Effect Transistors (MOSFETs) operating at high voltages (>3 kV). However, the fabrication of SJ devices presents significant challenges due to fabrication complexity. This paper presents a comprehensive analysis of a feasible and easy-to-fabricate semi-superjunction (SSJ) design for 3.3 kV SiC MOSFETs. The proposed approach utilizes trench etching and sidewall implantation, with a tilted trench to facilitate the implantation process. Through Technology Computer-Aided Design (TCAD) simulations, we investigate the effects of the *p*-type sidewall on the charge balance and how it affects key performance characteristics, such as breakdown voltage (BV) and on-state resistance (R_DS-ON_). In particular, both planar gate (PSSJ) and trench gate (TSSJ) designs are simulated to evaluate their performance improvements over conventional planar MOSFETs. The PSSJ design achieves a 2.5% increase in BV and a 48.7% reduction in R_DS-ON_, while the TSSJ design further optimizes these trade-offs, with a 3.1% improvement in BV and a significant 64.8% reduction in R_DS-ON_ compared to the benchmark. These results underscore the potential of tilted trench SSJ designs to significantly enhance the performance of SiC SSJ MOSFETs for high-voltage power electronics while simplifying fabrication and lowering costs.

## 1. Introduction

Silicon carbide (SiC) Metal Oxide Semiconductor Field-Effect Transistors (MOSFETs) offer several advantages over traditional silicon (Si) devices due to the superior properties of the material, including a higher breakdown electric field, superior thermal conductivity, faster switching speeds, and lower on-state resistance [1]. These advantages enable SiC MOSFETs to handle higher voltages and operate at higher frequencies with greater efficiency, making them ideal for high-performance power electronic systems [2,3,4]. However, despite their advantages, SiC MOSFETs face significant challenges when targeting voltage ratings above 3000 V. The primary reason for these issues is the thick drift region required for high voltage blocking in SiC devices, which increases both resistance and the complexity of fabrication. Therefore, silicon-based devices, such as Si IGBTs, continue to dominate this market segment due to the high conduction losses and increased fabrication costs of their SiC counterparts. As a result, there is a need for innovative design approaches that can maximize the inherent benefits of SiC while minimizing its limitations.

Superjunction (SJ) technology represents a promising approach to overcoming these challenges by surpassing the unipolar limit of SiC MOSFETs [5,6,7]. This technology improves the trade-off between conduction losses and breakdown voltage by alternating *p*-type and *n*-type pillars within the drift region. The fabrication of SiC SJ MOSFETs faces significant challenges, requiring advanced fabrication techniques and precise doping control to ensure optimal performance [8].

One of the main SJ fabrication methods is multi-epitaxial (ME) growth [9,10], which involves multiple cycles of epitaxial growth and ion implantation to create alternating *p*-pillars. While effective, this process is challenging for thick, deep structures due to the low diffusion coefficients of dopants and the inherent hardness of SiC, which complicates the fabrication and increases costs.

An alternative approach is trench-filling epitaxial (TFE) growth [11], which involves etching trenches into the *n*-type SiC substrate, refilling them with *p*-type epitaxial layers, and planarizing the surface using Chemical Mechanical Polishing (CMP). Although TFE growth offers more precise control over doping profiles, it also presents challenges in achieving uniform refill and comes with high fabrication costs.

To address these fabrication challenges while still harnessing the benefits of SJ technology, the semi-superjunction (SSJ) approach has emerged as a practical compromise [12,13,14]. Unlike full SJ designs, SSJ structures do not fully implement the superjunction architecture, which simplifies the fabrication process and reduces costs. Despite this simplification, SSJ designs can still provide significant performance improvements over traditional SiC MOSFETs, making them a valid option for high-voltage applications [15].

The authors previously introduced cost-effective methods for the fabrication of SJ Schottky Barrier Diodes (SBDs) using trench etching and sidewall implantations [16,17,18].

This paper presents, through a comprehensive numerical study, 3.3 kV SiC MOSFETs for a cost-effective and easy-to-fabricate design. The SJ effect is obtained through a process of trench etching and sidewall implantation, utilizing a tilted trench to simplify the fabrication process and improve manufacturability [19].

**Figure 1:**
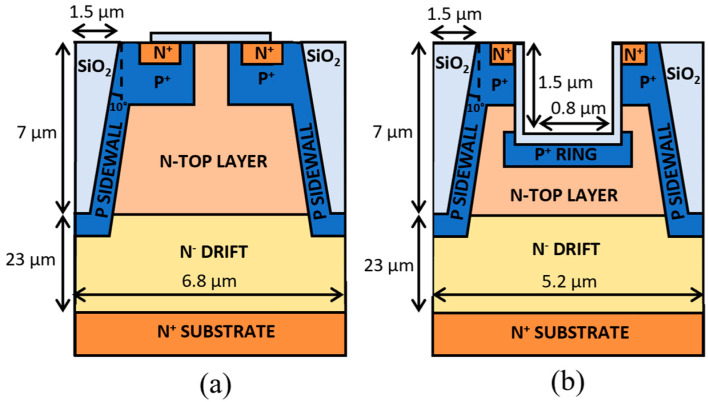
Sketch of the proposed devices. (**a**) Planar gate semi superjunction (PSSJ). (**b**) Trench gate semi superjunction (TSSJ). Not to scale.

Two different designs, sketched in Figure 1, are analyzed: a planar gate SSJ (PSSJ) (Figure 1a) and a trench gate SSJ (TSSJ) (Figure 1b). This study focuses on the static performance of the proposed designs, specifically analyzing breakdown voltage (BV), on-state resistance (R_DS-ON_), and electric field distribution. These parameters are evaluated using Technology Computer-Aided Design (TCAD) simulations performed with the finite element simulator Sentaurus, developed by Synopsys [20]. Although our study does not investigate switching behavior in detail [21,22], previous works [23] have shown that semi-SJ trench structures exhibit fast switching characteristics due to their majority-carrier nature. Given that the proposed SSJ MOSFETs operate under the same principle, a similar switching behavior is expected. Moreover, while we acknowledge the importance of self-heating [24,25], we do not focus on this phenomenon in our study. Our primary objective, however, is to compare the static performance of the proposed semi-superjunction (SSJ) designs against a standard design. The results are compared to current state-of-the-art devices at the 3.3 kV breakdown rating, aiming to demonstrate the potential of SSJ designs to optimize the performance and cost-effectiveness of high-voltage SiC MOSFETs.

## 2. Benchmark Structures

**Figure 2:**
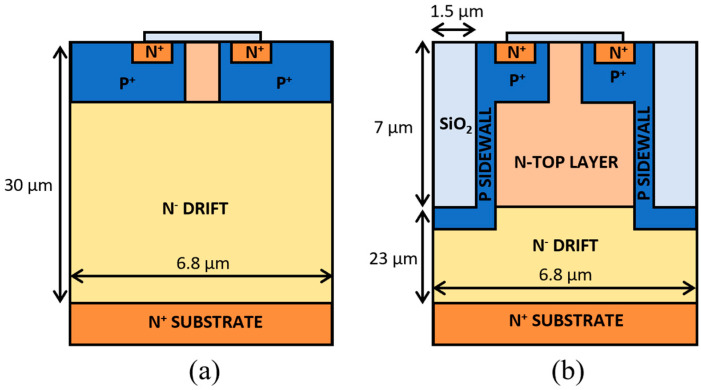
Sketch of the reference devices. (**a**) Planar (benchmark). (**b**) Vertical semi-superjunction (ideal). Not to scale.

To accurately evaluate the performance enhancements provided by the proposed tilted trench semi-superjunction designs, it is essential to establish benchmark structures for comparison. The benchmarks chosen for this study are a standard planar MOSFET and an ideal vertical SSJ MOSFET. The planar MOSFET serves as a primary benchmark structure due to its widespread use in power electronics and provides a reference for the performance of traditional SiC devices under similar conditions. The ideal vertical SSJ MOSFET represents the theoretical best-case scenario for trench etching and sidewall implantation SSJ devices, serving to benchmark the upper limits of performance achievable with this technology. The study of the ideal case allows an understanding of the maximum potential improvements possible with this SSJ approach and the determination of whether there is a sufficient margin to explore the more practical, yet non-ideal, tilted trench design. The benchmark structures are sketched in Figure 2.

All simulated structures in this work utilize a 4H-SiC substrate, which is 100 µm thick and has a constant nitrogen doping concentration of 1 × 10^19^ cm^−3^. To achieve a BV greater than 3.3 kV, the devices have a drift region thickness of 30 µm with a constant doping concentration of 3 × 10^15^ cm^−3^. The *n*+ source region has a peak doping concentration of 1 × 10^19^ cm^−3^ and the *p* contact for the body region has a constant doping concentration of 1 × 10^19^ cm^−3^ to ensure good ohmic contact. More specifically, a Gaussian doping profile is assumed for the body and source regions, reflecting the realistic diffusion and implantation processes commonly used during device fabrication. In contrast, the epitaxial layer and substrate are modeled with constant doping concentrations. This approximation is widely adopted in device simulations due to the high uniformity typically achieved in epitaxial growth processes and the homogeneous nature of substrate materials. To capture the transition between the epitaxial layer and the substrate accurately, a Gaussian transition doping profile is applied at their interface, ensuring a smooth change in doping concentration and preserving the physical accuracy of the model. A 50 nm thick gate oxide is considered, with a fixed charge at the gate oxide/semiconductor interface of 2 × 10^12^ cm^−3^ [26,27]. The area factor is adjusted to consider a total area of the simulated devices of 1 cm^2^.

### 2.1. Planar MOSFET

**Figure 3:**
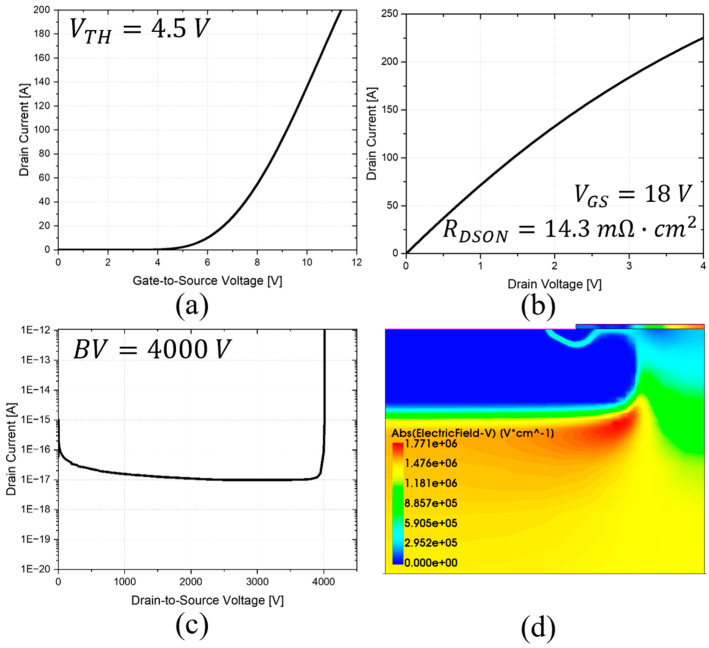
Planar structure performance. (**a**) Transfer characteristic. (**b**) On-state characteristic. (**c**) Off-state characteristic. (**d**) Electric field distribution at 2 kV.

A standard planar MOSFET (Figure 2a) was simulated with a cell pitch of 6.8 µm to maintain consistency with the other simulated structures. The threshold voltage (V_TH_) was around 4.5 V, as shown by the transfer characteristic in Figure 3a. A JFET width of 1.2 µm was considered, and a constant doping concentration of 6 × 10^15^ cm^−3^ was also included to improve the on-state conduction of the device [28]. The resulting output characteristic for a V_GS_ of 18 V is reported in Figure 3b. The corresponding R_DS-ON_ is 14.3 mΩ·cm^2^ [29]. The reverse characteristic is shown in Figure 3c, indicating a BV of 4000 V. The electric field (EF) distribution at 2 kV, reported in Figure 3d, shows that the electric field peak (1.7 MV/cm) is located at the bottom of the body region, while the gate oxide is free from EF pressure.

### 2.2. Vertical Semi-Superjunction MOSFET

To achieve a uniform electric field distribution in the superjunction designs, a charge balance between the *n*-layer and the *p*-type sidewall implantation should be attained [30] (see Equation (1)).

(1) $$Q_{N}=Q_{P}$$

This balance can be obtained following the formula reported in Equation (2).

(2) $$N_{D}W_{N}=N_{A}W_{P}$$

where N_D_ and W_N_ are the doping concentration and width of the *n*-layer, respectively, and N_A_ and W_P_ are the doping concentration and width of the *p*-type sidewall, respectively. According to Equation (2), the ideal semi-superjunction is achieved when the trench angle is 0 degrees, resulting in a constant W_N_ along the trench and thus achieving a fully compensated charge between the *p*-type sidewall and the contiguous *n*-zone. This 0-degree trench configuration allows for the best performance with this design.

A vertical SSJ with trench etching and sidewall implantation was simulated as an ideal case. The design featured an oxide-filled trench 3 µm wide and 7 µm deep. A sidewall *p*-type implantation was considered to allow the SJ effect. The *p*-doping profiles along the sidewalls were modeled with Gaussian distributions to accurately reflect realistic implantation and diffusion processes. A fixed charge of 5 × 10^12^ cm^−3^ at the interface between the SJ trench and the semiconductor was considered [31] to take into consideration additional edge roughness. All other design parameters were consistent with those of the planar MOSFET. To ensure the charge balance, an *n*-top layer with a doping concentration (N_TOP_) higher than the drift region was considered next to the oxide trench.

**Figure 4:**
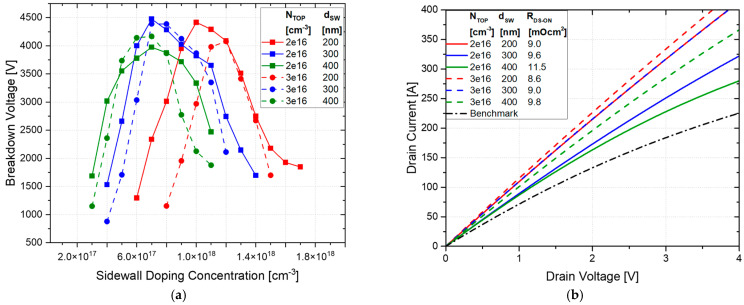
Vertical SSJ performance. (**a**) Analysis of the breakdown voltage versus sidewall peak doping concentration, varying N_TOP_ (solid line 2 × 10^16^ cm^−3^, dotted line 3 × 10^16^ cm^−3^) and sidewall depth (red 200 nm, blue 300 nm, green 400 nm) for the vertical SSJ. (**b**) On- state characteristics and R_DS-ON_, varying N_TOP_ and sidewall depth for the vertical SSJ. The sidewall doping concentration is set to the values that provide the best charge balance for each combination of N_TOP_ and d_SW_.

An analysis of its performance was conducted by varying the *p*-sidewall peak doping concentration and implantation depth (d_SW_). Variations in the JFET *n*-top layer doping concentrations of 2 × 10^16^ cm^−3^ and 3 × 10^16^ cm^−3^, as well as *p*-sidewall depths of 200 nm to 400 nm, were considered. The resulting behavior of the BV is reported in Figure 4a.

A wide implantation window was obtained for most of the design parameter combinations, improving the BV by up to 11.9% compared to the planar case.

**Figure 5:**
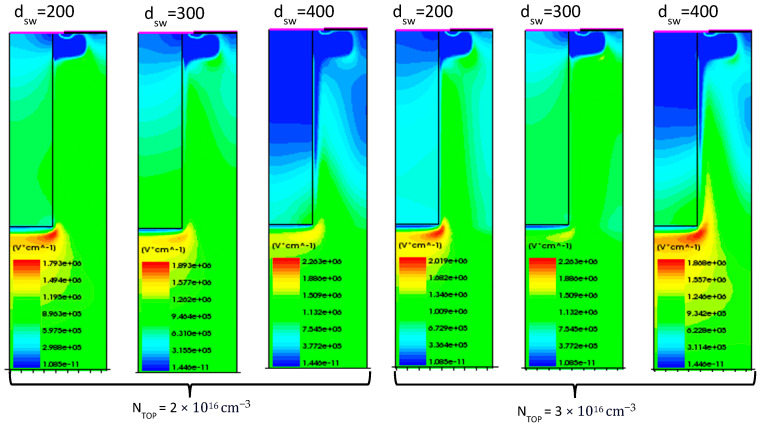
Electric field distribution at 2 kV, varying N_TOP_ and sidewall depth for the ideal vertical SSJ. The sidewall doping concentration corresponds to the optimal BV value for each case.

A significant improvement in the on-state conduction was also achieved, with R_DS-ON_ varying from 8.6 mΩ·cm^2^ to 11.5 mΩ·cm^2^, ensuring an improvement from 21.7% to 49.8%, as shown in Figure 4b. The on-state characteristics were simulated using the *p*-sidewall concentration that achieved the optimal charge balance for each combination of N-top layer doping and sidewall depth, according to Figure 4a. In Figure 5, the EF distribution at 2 kV is reported, showing reasonable values for the EF peak value.

Despite significantly improved performance over the planar design, a 0-degree trench posed significant challenges for the implantation through the sidewalls. However, the promising results obtained from the ideal vertical SSJ case indicate that substantial performance enhancements are achievable through this technology. This suggests that there is a sufficient margin to explore the tilted trench design, which, although non-ideal, may offer a practical compromise between performance, fabrication feasibility, and cost.

### 2.3. Tilted Semi-Superjunction MOSFET

To overcome the challenges associated with implantation through a vertical sidewall and make the implantation process more feasible, a tilted trench with an angle of 10 degrees was considered, pivoting at the midpoint of the trench depth (i.e., 3.5 µm). This inclination facilitates the implantation process by providing better access to the trench sidewalls, reducing the difficulty of achieving uniform doping profiles. The details of the fabrication of SiC superjunction trenches have already been discussed by the authors in [32].

In an ideal vertical superjunction structure, the width of the *n*-type region (W_N_) would remain constant along the trench depth to ensure uniform charge compensation between the *p*- and *n*-type regions. In the tilted trench design, the trench wall slope causes a variation in W_N_ along the trench depth, which affects the charge distribution. Specifically, when the *n*-top layer doping concentration is optimized to balance charges at the midpoint of the trench, this results in an overcompensation of positive charges (Q_P_) above the pivot point and an overcompensation of negative charges (Q_N_) below the pivot point. This uneven charge distribution can lead to localized electric field peaks, potentially increasing the risk of premature breakdown or affecting the device’s breakdown voltage.

The impact of this charge imbalance is further exacerbated at different depths of the trench. Above the pivot point, where Q_P_ is overcompensated, the electric field may be more concentrated, leading to higher field stress in these regions. Conversely, below the pivot point, the overcompensation of Q_N_ can create regions with a lower electric field intensity, which might reduce the overall effectiveness of the SJ effect. Therefore, while the tilted trench design simplifies fabrication, it necessitates the careful optimization of doping profiles and trench geometry to balance these competing effects and achieve the desired device performance.

An analysis of the performance of the proposed PSSJ and TSSJ designs is reported below.

Firstly, a planar channel semi-superjunction design with a tilted oxide trench (see Figure 1) is simulated, focusing on optimizing the SSJ effect while minimizing charge imbalance. To further exploit the SSJ effect, a TSSJ design is considered, incorporating a trench gate that is 0.8 µm wide and 1.5 µm deep. The absence of a JFET region in this design allows for a reduced cell pitch of 5.2 µm. Additionally, a *p*-ring with a high doping concentration of 5 × 10^18^ cm^−3^ is added at the bottom of the trench to shield the gate oxide from excessive electric fields, enhancing device reliability.

The analysis includes variations in sidewall doping concentration across different sidewall depths (200 nm, 300 nm, 400 nm) and different doping concentrations for the JFET *n*-top layer (2 × 10^16^ cm^−3^ and 3 × 10^16^ cm^−3^), aiming to optimize the overall device performance in terms of BV and R_DS-ON_. An overview of the doping concentrations and dimensions of the simulated structures is provided in Table 1.

## 3. Results and Discussion

The performance of the proposed designs in terms of BV, EF distribution, and R_DS-ON_ was evaluated through TCAD simulations. These simulations provide insights into how varying design parameters, such as doping concentrations and trench geometry, influence the electrical characteristics of planar and trench SSJ MOSFETs.

### 3.1. Planar Semi-Superjunction

**Figure 6:**
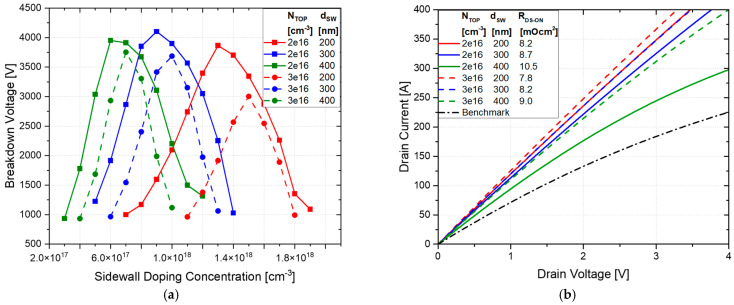
PSSJ performance. (**a**) Analysis of the breakdown voltage versus the sidewall peak doping concentration with varying N_TOP_ (solid line 2 × 10^16^ cm^−3^, dotted line 3 × 10^16^ cm^−3^) and sidewall depth (red 200 nm, blue 300 nm, green 400 nm) values for the PSSJ. (**b**) On-state characteristics and R_DS-ON_, varying N_TOP_ and sidewall depth for the PSSJ. The sidewall doping concentration is set to the values that provide the best charge balance for each combination of N_TOP_ and d_SW_.

The behavior of the BV as a function of the sidewall peak doping concentration is reported in Figure 6a. The maximum achievable BV is 4101 V, obtained with an *n*-top layer doping concentration of 2 × 10^16^ cm^−3^ and a sidewall doping concentration and depth of 9 × 10^17^ cm^−3^ and 300 nm, respectively. For a JFET *n*-layer doping concentration of 2 × 10^16^ cm^−3^, the BV across all sidewall depths approaches and indeed exceeds the benchmark value (4 kV) but remains below the ideal SSJ case (4.4 kV). This trend suggests that while the doping concentration is sufficient to establish a good electric field distribution, it does not fully optimize charge balancing. For a higher JFET *n*-top layer doping concentration (i.e., 3 × 10^16^ cm^−3^), the BV is significantly lower. This decrease can be attributed to an increased imbalance in the charge distribution between the *n*- and *p*-type regions, resulting in localized electric field enhancements that reduce the overall breakdown voltage.

Conversely, the lowest BV is observed for an *n*-top layer doping concentration of 3 × 10^16^ cm^−3^ with a sidewall depth of 200 nm as the high *n*-layer concentration and shallow *p*-sidewall result in maximum charge imbalance. This imbalance significantly reduces the effectiveness of charge compensation, leading to a BV of 3 kV.

Figure 6b shows the on-state characteristics and the relative R_DS-ON_ values when varying the *n*-top layer doping concentration and sidewall depth. It is worth noting that the on-state performance is not significantly affected by the *p*-doped sidewall doping concentration. The R_DS-ON_ decreases as the *n*-top layer doping concentration increases, whereas it increases when the sidewall depth decreases. This trend occurs because a higher doping concentration increases the number of charge carriers, reducing resistance, whereas a shallower sidewall enlarges the effective current conduction path, thereby reducing the overall resistance.

The best result is achieved with an *n*-top layer doping concentration of 3 × 10^16^ cm^−3^ and a sidewall depth of 200 nm, yielding an R_DS-ON_ of 7.8 mΩ·cm^2^. This configuration provides an effective conduction path that minimizes resistance. Conversely, the least efficient result is observed for a constant *n*-top layer doping concentration of 2 × 10^16^ cm^−3^ with a sidewall depth of 400 nm, resulting in an R_DS-ON_ of 10.5 mΩ·cm^2^. The deeper sidewall and lower doping concentration reduce the effective carrier density and cause a narrower conduction path, increasing resistance.

**Figure 7:**
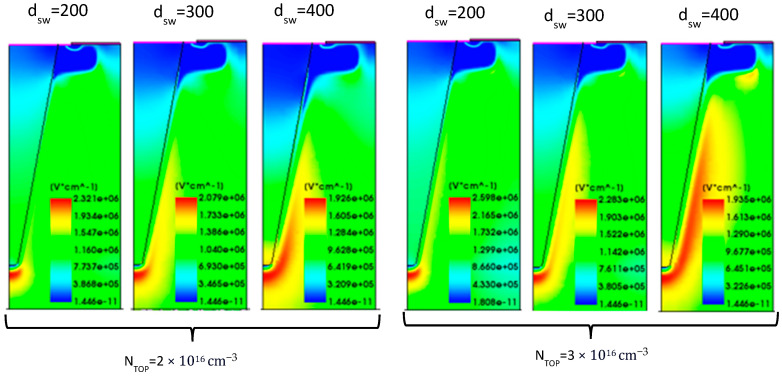
Electric field distribution at 2 kV, varying the N_TOP_ and sidewall depth for the PSSJ. The sidewall doping concentration corresponds to the optimal BV value for each case.

Figure 7 shows the electric field distribution at 2 kV for the optimal breakdown voltage corresponding to each combination of sidewall depth and *n*-top layer doping concentration. A deeper sidewall doping leads to a more uniform EF distribution along the oxide trench while also reducing the peak electric field. This uniformity is critical for enhancing device reliability, as it minimizes the likelihood of field-induced failures.

Table 2 reports an overview of the performance of this planar semi-superjunction design, including percentage variations relative to the benchmark planar structure. The optimal trade-off is observed in Case 2, with a BV of 4101 V and an R_DS-ON_ of 8.7 mΩ·cm^2^, improving the performance of the benchmark by 2.5% and 48.7%, respectively.

### 3.2. Trench Semi-Superjunction

**Figure 8:**
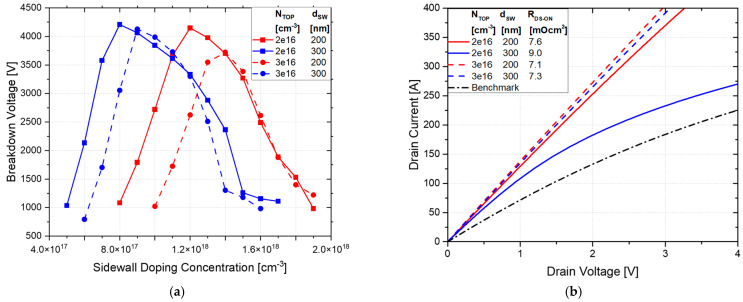
TSSJ performance. (**a**) Analysis of the breakdown voltage versus sidewall peak doping concentration, varying N_TOP_ (solid line 2 × 10^16^ cm^−3^, dotted line 3 × 10^16^ cm^−3^) and sidewall depth (red 200 nm, blue 300 nm, green 400 nm) for the vertical SSJ. (**b**) On-state characteristics and R_DS-ON_, varying N_TOP_ and sidewall depth for the TSSJ. The sidewall doping concentration is set to the values that provide the best charge balance for each combination of N_TOP_ and d_SW_.

The BV versus the sidewall peak doping concentration for the trench gate semi-superjunction design is reported in Figure 8a. Similar trends to the planar structure are observed, but the reduced pitch in the trench design enables higher BV values across a broader range of design parameter combinations. More specifically, the benchmark values are exceeded for most parameter combinations. The best BV value occurs again at a doping concentration of 2 × 10^16^ cm^−3^ with a sidewall depth of 300 nm, resulting in a BV of 4206 V, which improves the value of the benchmark by 5.0%.

**Figure 9:**
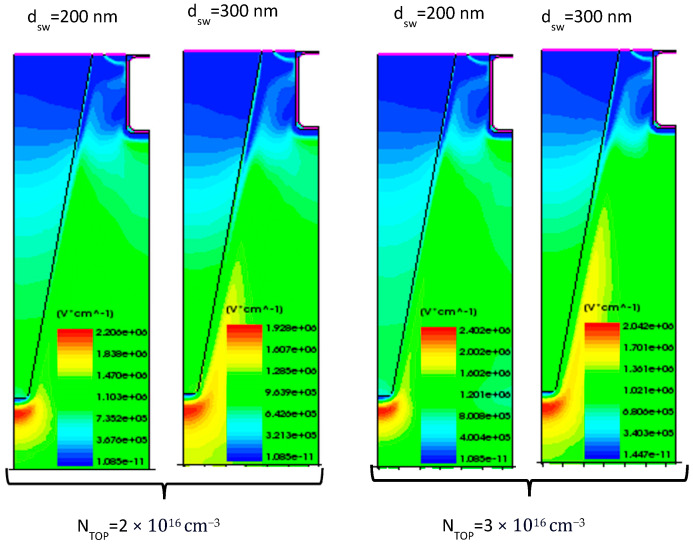
Electric field distribution at 2 kV, varying N_TOP_ and sidewall depth for the TSSJ. The sidewall doping concentration corresponds to the optimal BV value for each case.

The reduced pitch between the trenches further enhances electric field management, allowing for a higher BV. The reduction in SJ *n*-pillar width allows for better SJ charge balancing (towards the bottom of the SJ trench). The incorporation of a *p*-well below the gate trench shifts the EF peak away from the oxide, enhancing gate reliability and reducing the risk of oxide breakdown. As a result, the smaller cell pitch reduces the maximum EF compared to the PSSJ structure, further improving the electric field profile at 2 kV, as shown in Figure 9.

The trench design also significantly enhances on-state conduction in certain cases, achieving much better performance compared to the standard planar and planar gate SSJ structures. Figure 8b presents the on-state characteristics and the corresponding R_DS-ON_. The lowest R_DS-ON_ value is 7.1 mΩ·cm^2^ (67.3% lower than the benchmark), obtained with a doping concentration of the *n*-top layer of 3 × 10^16^ cm^−3^ and sidewall depth of 200 nm.

**Figure 10:**
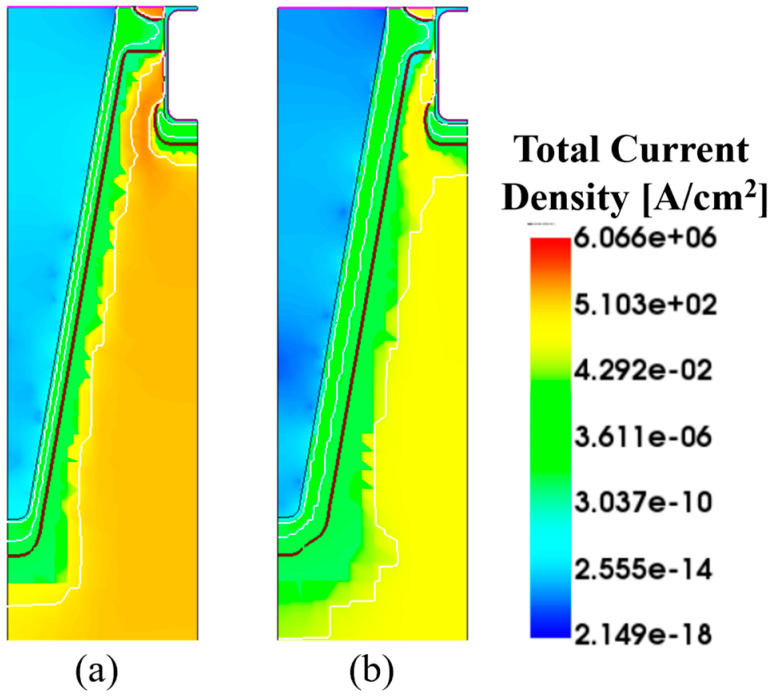
Total current density path for an *n*-top layer doping concentration of 3 × 10^16^ cm^−3^ and (**a**) d_SW_ = 200 nm; and (**b**) d_SW_ = 400 nm. In the figure is highlighted the junction line (black lines).

For lower *n*-top layer doping concentrations (e.g., 2 × 10^16^ cm^−3^), the depletion region becomes excessively large for deeper sidewall dopings, hindering current conduction between the gate trench and the deep SJ trenches. In other words, the current path between the *p*-sidewall and the *p*-ring area becomes very narrow, obstructing the current flow, as shown in Figure 10. This narrow path limits carrier mobility and increases resistance. Thus, optimizing the trench width and sidewall depth is crucial for balancing R_DS-ON_ and BV. According to this, a sidewall doping depth of 400 nm is not considered for this design to prevent worsening conduction performance. The cell pitch is determined according to the resulting JFET resistance, ensuring that the trench dimensions support efficient conduction without compromising breakdown performance. A summary of the performance of the trench structure is provided in Table 3. The best trade-off for the trench gate design is obtained in Case 4, with a BV of 4127 V and an R_DS-ON_ of 7.3 mΩ·cm^2^, improving the benchmark by 3.1% and 64.8%, respectively.

### 3.3. The Graded Approach

As the width of the *n*-SJ layer near the trench varies due to the trench inclination, to perfectly balance the charge alongside the tilted trench, the *n*-top layer doping concentration should decrease as the *n*-width increases to maintain a constant charge density all across. This approach addresses the issue of unbalanced charges along the tilted trench by compensating for the varying widths of the *n*-region. In areas where the *n*-width is greater, the doping concentration is reduced to prevent excessive charge accumulation, while in narrower sections, the *n*-doping concentration is increased to ensure sufficient charge balance.

A simple and feasible approach to achieve this graded doping profile is to divide the *n*-top layer into several sections, each with a different doping concentration. This method is straightforward because different doping profiles can be easily achieved through multiple epitaxial growth steps at the initial drift epi growth stage, allowing for more precise control of the charge distribution between the *p*- and *n*-regions.

By tailoring the doping levels to the varying widths of the *n*-region, the graded approach can effectively balance the charge, enhancing the overall performance and reliability of the tilted trench SSJ design.

**Figure 11:**
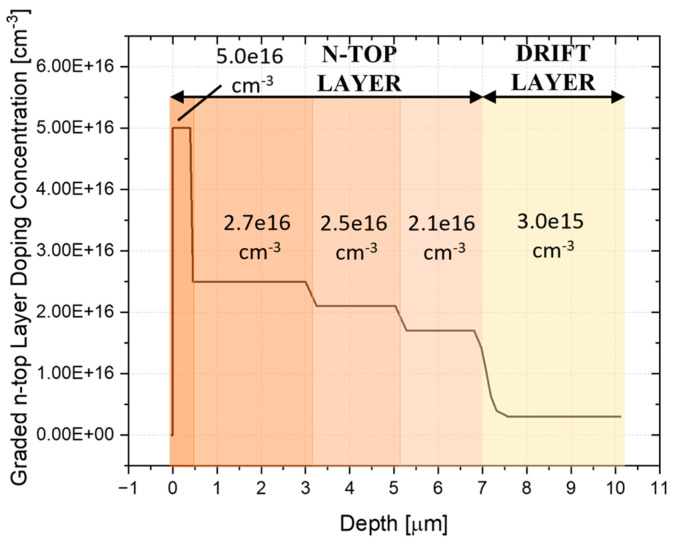
Graded *n*-top layer doping concentration.

To explore the effectiveness of this graded approach, a simulation was performed on the PSSJ considering a sidewall depth of 300 nm and a peak *p*-doping concentration of 1 × 10^18^ cm^−3^, which were found to be the optimal parameters for the constant *n*-top layer case. The *n*-top layer was divided into four sections, with doping concentrations of 5.0 × 10^16^ cm^−3^, 2.5 × 10^16^ cm^−3^, 2.1 × 10^16^ cm^−3^, and 1.7 × 10^16^ cm^−3^. The resulting doping profile is reported in Figure 11.

The simulation showed a BV of 4055 V and an R_DS-ON_ of 8.2 mΩ·cm^2^. Therefore, the BV remains high (>4 kV), and the graded approach results in a further 6% improvement in R_DS-ON_. An overview of the performance of all the analyzed designs is reported in Table 4. These results suggest that there is potential for further optimization by tuning the number of zones, their extension, and their doping concentration.

However, the graded approach might not be suitable for trench designs due to the presence of the *p*-ring, an external element that further disturbs charge balance. The *p*-ring complicates the implementation of a precise doping gradient, as it introduces additional variability in the electric field and charge distribution. Consequently, while the graded approach shows promise for the PSSJ design, its applicability to trench-based designs may be limited.

## 4. Conclusions

This paper presents a detailed analysis of 3.3 kV SiC MOSFETs utilizing a tilted trench semi-superjunction design, demonstrating a cost-effective approach to improving device performance. Through TCAD simulations, we demonstrated the advantages of this design over conventional planar structures, showing an enhanced breakdown voltage and reduced on-state resistance. For the planar gate SSJ design, the best trade-off between on-state and blocking performance was achieved with a *p*-sidewall doping depth of 300 nm and a JFET *n*-top layer concentration of 2 × 10^16^ cm^−3^, resulting in improvements of 2.5% in the BV and 48.7% in the R_DS-ON_ compared to the benchmark. In the trench gate SSJ design, the trade-off was further optimized, with improvements over the benchmark of 3.1% for BV and 64.8% for R_DS-ON_.

Additionally, the graded approach was explored by dividing the *n*-top layer into four zones, each with a different constant doping concentration. This method provided a further improvement of 6% in R_DS-ON_ for the planar gate design without adding any significant fabrication complexity. These findings suggest that tilted trench SSJ designs offer a promising strategy for enhancing the performance and reliability of SiC MOSFETs, making them well suited for demanding power electronic applications.

## Figures and Tables

**Table 1:** Overview of the doping concentrations and dimensions of the simulated structures.

	Value				Unit
	Planar	Vertical SSJ	PSSJ	TSSJ	
Drift doping	3 × 10^15^				cm^−3^
Body doping	1 × 10^19^				
Source doping	1 × 10^19^				
Substrate doping	1 × 10^19^				
*N*-top layer doping	/	2 × 10^16^, 3 × 10^16^			
*P*-sidewall peak doping	/	2 × 10^17^ − 2 × 10^18^			
Drift thickness	30				μm
Substrate thickness	100				
Cell pitch	6.8			5.2	
SJ trench depth	/	7			
SJ trench width	/	3			
*P*-sidewall depth	/	0.2, 0.3, 0.4		0.2, 0.3	
Gate trench width	/			0.8	
Gate trench depth	/			1.5	
SJ trench angle	/	0	10		°

**Table 2:** Performance overview of the PSSJ and percentage variation relative to the benchmark.

Case	*N*-Top Layer Doping Concentration [cm^−3^]	d_SW_ [nm]	BV [V]	R_DS-ON_ [mΩ·cm^2^]
1	2 × 10^16^	200	3864 (−3.4%)	8.2 (−54.2%)
2		300	4101 (+2.5%)	8.7 (−48.7%)
3		400	3951 (−1.2%)	10.5 (−30.6%)
4	3 × 10^16^	200	3003 (−28.7%)	7.8 (−58.8%)
5		300	3686 (−8.1%)	8.2 (−54.2%)
6		400	3752 (−6.4%)	9.0 (−45.5%)

**Table 3:** Performance overview of the TSSJ and percentage variation relative to the benchmark.

Case	*N*-Top Layer Doping Concentration [cm^−3^]	d_SW_ [nm]	BV [V]	R_DS-ON_ [mΩ·cm^2^]
1	2 × 10^16^	200	4148 (+3.6%)	7.6 (−61.2%)
2		300	4206 (+5.0%)	9.0 (−45.5%)
3	3 × 10^16^	200	3722 (−7.2%)	7.1 (−67.3%)
4		300	4127 (+3.1%)	7.3 (−64.8%)

**Table 4:** General performance overview and percentage variation relative to the benchmark.

Design	BV [V]	R_DS-ON_ [mΩ·cm^2^]
Benchmark	4000 (−)	14.3 (−)
PSSJ	4101 (+2.5%)	8.7 (−48.7%)
TSSJ	4127 (+3.1%)	7.3 (−64.8%)
Graded PSSJ	4055 (+1.4%)	8.2 (−54.2%)

## Data Availability

The original contributions presented in the study are included in the article, further inquiries can be directed to the corresponding author.
